# Sequential Treatment of Biliary Atresia With Kasai Hepatoportoenterostomy and Liver Transplantation: Benefits, Risks, and Outcome in 393 Children

**DOI:** 10.3389/fped.2021.697581

**Published:** 2021-07-07

**Authors:** Roberto Tambucci, Catherine de Magnée, Margot Szabo, Aniss Channaoui, Aurore Pire, Vanessa de Meester de Betzenbroeck, Isabelle Scheers, Xavier Stephenne, Françoise Smets, Etienne M. Sokal, Raymond Reding

**Affiliations:** ^1^Pediatric Surgery and Transplantation Unit, Department of Surgery, Cliniques Universitaires Saint-Luc, Université Catholique de Louvain, Brussels, Belgium; ^2^Pediatric Gastroenterology and Hepatology Division, Department of Pediatrics, Cliniques Universitaires Saint-Luc, Université Catholique de Louvain, Brussels, Belgium

**Keywords:** biliary atresia, Kasai procedure, pediatric liver transplantation, PELD score, patient survival rate, transplant surgical complications, graft survival rate, hepatoportoenterostomy

## Abstract

**Introduction:** Surgical treatment of biliary atresia (BA) is still based on sequential strategy with Kasai hepatoportoenterostomy (KP) followed by liver transplantation (LT), in case of complicated secondary biliary cirrhosis. Concerns have been expressed regarding the risks of LT related to previous KP, suggesting primary LT as an exclusive treatment of BA.

**Methods:** Single-center retrospective analysis including 393 pediatric patients who underwent LT for BA from 1993 to 2018, categorized into two groups: with (KP) or without (NoKP) previous KP. Pre-LT clinical condition was estimated considering age at LT, time on waiting list, pediatric end-stage liver disease score (PELD), and presence of portal vein hypoplasia. Post-LT outcome was evaluated considering patient and graft survival rates, and need for early reoperation due to abdominal or graft-related complications (<45 days after LT).

**Results:** Two-hundred ninety-six patients (75.3%) were categorized in the KP group, and 97 (24.7%) in the NoKP group. Median age at LT was 1.14 years in the KP group and 0.85 years in the NoKP group (*p* < 0.0001). PELD score was significantly less severe in KP patients (*p* < 0.05). One-year patient survival rates were 96.9 and 96.8% in the KP and NoKP groups, respectively (*p* = 0.43), and the corresponding graft survival was 92.5 and 94.8% (*p* = 0.97). The need for early reoperation was more frequent in the KP group (29.8%) vs. NoKP group (12.4%, *p* = 0.01). The rate of bowel perforation was non-significantly higher in the KP group (8.1%) vs. NoKP group (3.1%, *p* = 0.11).

**Conclusions:** The sequential strategy including KP and LT allowed performing LT in patients with significant older age and better clinical conditions, when compared to those transplanted without previous KP. Patient and graft survivals were not impacted by previous KP. Although previous KP was associated with an increased rate of post-LT surgical complications, bowel perforation and bleeding did not occur significantly more frequently. Such results support the current strategy based on sequential treatment.

## Introduction

Biliary atresia (BA) is the most frequent cause of chronic cholestasis in infants, its incidence ranging from 1:5–10,000 in Asia to 1:15–20,000 live newborns in Western countries (1–4). In this condition, intrahepatic and/or extrahepatic inflammatory cholangiopathy of still uncertain pathophysiology leads to biliary duct obliteration during the first weeks of life, progressive liver fibrosis, secondary biliary cirrhosis, and eventually death, if not treated in a timely fashion (5, 6). Current treatments of BA have been introduced within a few years' interval around the 60s, when two surgical procedures were successively proposed: hepatoportoenterostomy by Morio Kasai in 1959 as an early approach to restore biliary drainage (7) and orthotopic liver transplantation (LT) by Thomas Starzl in 1963 for later management of end-stage biliary cirrhosis (8). Sixty years later, these two procedures remain the cornerstones of therapeutic options, but the benefit/risk ratio of Kasai procedure (KP) in this therapeutic sequence is still debated (9, 10). The growing experience in pediatric LT and particularly the technical difficulties encountered at LT following a previous KP led several authors to argue against the performance of KP, and for primary LT when indicated (11). Nevertheless, today, a strategy consisting in sequential KP followed by LT if required still constitutes the preferred scenario in most centers, particularly because KP allows 14–44% of BA patients to escape LT until adulthood (12–17). Paradoxically, only few studies have tried to systematically investigate whether LT outcomes are actually impacted by a previous KP, providing contradictory conclusions (18–23).

The aim of the present study was to document the possible impact of a previous hepatoportoenterostomy in a large LT series for BA, testing particularly the positive and negative consequences of KP on the clinical status at LT, as well as on post-LT overall outcome and surgical complications.

## Patients and Methods

### Study Design

A retrospective chart review was conducted on 393 consecutive pediatric BA patients who underwent LT at Cliniques Universitaires Saint-Luc, Université catholique de Louvain, Brussels, Belgium, between March 1, 1993, beginning of the living donor liver transplantation (LDLT) program at our institution, and December 31, 2018. This study was approved by the Institutional Review Board (Approval number: 2016/15NOV/491). The diagnosis of BA was done at intra-operative cholangiography and liver biopsy during KP, if performed, and confirmed at the pathological analysis of the hepatectomy specimen at LT. This series of LT for BA represented 53.5% of a total of 734 LT cases performed during the same time interval at our Institution. For the study purposes, BA patients were categorized in two groups, with or without KP prior to LT (KP group and NoKP group, respectively). These main groups were further stratified in subgroups based on the age at LT (<1, 1–2, 2–3, and >3 years). Preoperative data collected included age at KP, if done; age at LT; presence of growth retardation, defined as *z*-score for weight and/or height <2.0 SD; presence of polysplenia syndrome; waiting time on the deceased donor list; pediatric end-stage liver disease (PELD) score (24) calculated at time of LT, computing total serum bilirubin level, serum albumin, international normalized ratio (INR), and presence of growth retardation; and detection of portal hypoplasia, defined as a portal vein diameter ≤4 mm at LT (25). Intra-operative and postoperative outcome data collected included type of liver graft (deceased vs. living donor graft); overall patient and graft survival rates; causes of death; indications for re-transplantation; early mortality (within 1 year after LT); early surgical complications (redo surgery within 45 days after LT), including abdominal complications (bleeding, bowel perforation, bowel occlusion, and others) and graft complications (hepatic artery related, portal vein related, outflow blockage, biliary leakage and biliary anastomotic strictures); and overall long-term surgical graft complications (hepatic artery thrombosis, portal vein thrombosis, outflow blockage, biliary anastomotic strictures, biliary non-anastomotic strictures). Patients are eventually grouped in three different transplant eras, based on the date of LT: Era I (1993–1999), Era II (2000–2009), and Era III (2010–2018).

### Clinical Management

All patients were considered for KP as the first surgical option, in case of neonatal cholestasis detected before the age of 4 months and in the absence of advanced liver fibrosis/cirrhosis with signs of portal hypertension. Whatever the previous surgical status, LT was proposed in BA cases with persisting/worsening jaundice, and/or ascites, and/or recurring cholangitis, and/or gastrointestinal bleeding secondary to portal hypertension, and/or hepatopulmonary syndrome, and/or suspicion of secondary hepatocarcinoma (26). After full medical pre-LT workup, including assessment of liver function and nutritional statuses, as well as detailed Doppler-ultrasound analysis of hepatic parenchyma and hemodynamics, hypercaloric enteral feeding, fat-soluble vitamin supplementations, and parenteral nutrition were administered to the candidate recipient, when appropriate. Decompensated ascites was managed using diuretics and albumin infusion. Gastrointestinal hemorrhagic episodes were managed by correcting coagulopathy using vitamin K, fibrinogen, and plasma supplementation, as well as somatostatin analog infusion, when necessary. Variceal esophageal bleeding was treated endoscopically, using elastic banding or sclerotherapy. When multidisciplinary decision for LT was taken, all patients were registered on the EuroTransplant waiting list for deceased donor graft, if legally allowed according to the country of origin. LDLT was considered for patients in which deceased donor graft was not available, for those for whom any potential supplementary risk was estimated, or when the parents wished so. All intra-familial living donor candidates were carefully evaluated, with the aim to assess their health status, as well as to examine liver vascular/biliary anatomy and the volume of the left hepatic lobe (27). Overall management and surgical technique for LT have been detailed in previous reports from our group (27–31). For BA patients, some specific surgical techniques have been adopted during the study period. Preexisting Roux-en-Y jejunal loops from previous KP were systematically explored and, only if adequate length (at least 40 cm) and quality were evidenced, used as such for bilio-digestive reconstruction. When a previous Kasai loop was found to be too short (<40 cm), the termino-lateral jejuno-jejunal anastomosis was displaced more distally, to increase the loop length up to 50–60 cm. Management of portal vein hypoplasia was adapted based on anatomic presentation and types of donor and has been described in previous reports from our Institution (25, 27, 31); longitudinal portoplasty using inferior mesenteric vein patch was standardized as preferred techniques in LDLT; implantation of a long graft's portal trunk on the recipient's spleno-mesenteric venous confluence was used in case of deceased donor LT, when possible; and jump graft using autologous or heterologous venous graft implanted on the superior mesenteric vein has been widely used in the first period of this series and then progressively limited to selected cases. Doppler ultrasound has been systematically used intraoperatively and postoperatively, in order to detect any vascular, biliary, or other abdominal complications. Hepatic artery dysfunction or thrombosis was managed surgically if occurred within the first 14 days after LT, with the aim to restore adequate arterial flow, as recently reported (32). Similarly, any early portal vein dysfunction was treated medically, radiologically, or surgically; meso-Rex shunt was indicated in case of permanent portal vein thrombosis (33, 34). Hepatic outflow dysfunction was detected and managed by ultrasound and interventional radiology, while surgery was indicated in very selected cases. Biliary leakage was managed conservatively, when possible, in the absence of signs of peritonitis. Anastomotic biliary strictures were preferentially treated by surgery, while interventional radiology was considered in selected cases (35). Non-anastomotic biliary strictures were managed by interventional radiology, and re-transplantation was considered in case of secondary biliary cirrhosis and/or biliary infection/sepsis. Peritonitis due to bowel perforation was managed with surgical re-exploration, and diverting enterostomy was often considered, based on intraoperative findings. Post-LT immunosuppressive protocols varied along these last 25 years and have been already documented in the literature (36, 37).

### Statistical Analysis

With the aim to assess the impact of previous Kasai operation, patients in the KP group were compared to those in the NoKP group for any of the abovementioned variables (as shown on the right side of each rows in the tables). Continuous variables were studied using the Mann–Whitney test, one-way ANOVA, and log-rank analysis. Results are expressed as median and interquartile range (IQR). Discrete variables were studied using Fisher's exact test and chi-square test and are expressed as number and percentage, or as median and IQR, as appropriate. Since patients in the NoKP group were transplanted earlier in life, while those in the KP group were transplanted within a larger age range (up to more than 15 years), comparison analysis was also stratified for age subgroups (<1, 1–2, 2–3, >3 years). For each separate group (KP/NoKP), data from age subgroups were further compared to assess the possible trend of variation according to the age at LT (as shown on the bottom of columns in the tables). This analysis was done using the ANOVA test for trend, chi-square test for trend, and log-rank test for trend, as appropriate. The correlation between age at LT and pre-LT variables was also analyzed using linear regression analysis, including only patients <3 years old. Finally, analyses were repeated comparing patients (overall, KP group and NoKp group separately) according to transplant eras. Differences were considered statistically significant when the *p*-value (two-tailed) was <0.05. Statistical analysis was performed using the software GraphPad Prism (version 8.4.3 for Mac, GraphPad Software, San Diego, CA, USA, www.graphpad.com).

## Results

### Distribution of Patients

**Figure 1:**
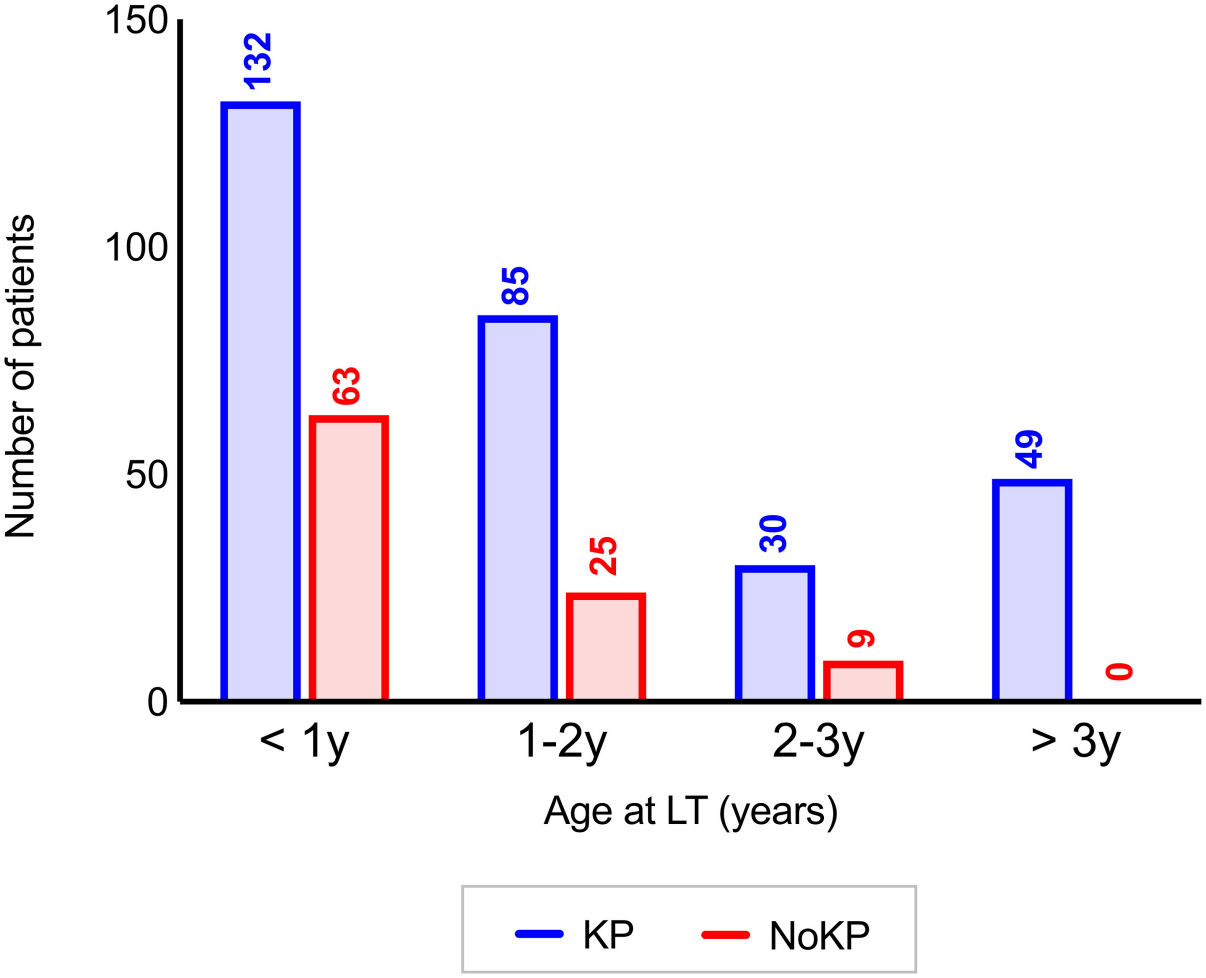
Distribution of 393 patients in the KP and NoKP groups according to the age at LT (*p*-value for trend <0.0001). KP, Kasai procedure (patient with previous Kasai operation); NoKP, No Kasai procedure (patients without previous Kasai operation); LT, liver transplantation.

Among the 393 transplanted patients for BA, 296 (75.3%) had undergone KP before LT (KP group), while 97 (24.7%) received LT without previous KP (NoKP group). One hundred ninety-five patients (49.6%) were transplanted before the age of 1 year (132 in the KP group vs. 63 in the NoKP group), 110 (28.0%) between the age of 1 and 2 years (85 KP vs. 25 in NoKP), 39 (9.9%) between the age of 2 and 3 years (30 KP and 9 No KP), and 49 (12.5%) between 3 and 16 years (49 KP and 0 NoKP) (*p*-value for trend < 0.0001) (Figure 1). The median age at the time of Kasai operation was 8 weeks (IQR 6–10; range 1–20) and was not significantly different among age subgroups at LT (*p* = 0.27).

### Pre-transplantation Clinical Conditions

Pre-LT clinical conditions variables are summarized in (Table 1). Age at LT was significantly lower in patients in the NoKP group (*p* < 0.0001). Interestingly, however, the youngest patient of the whole series belonged to the KP group (0.3 years). No patient in the NoKP group was transplanted beyond the age of 3 years. Similarly, time on the waiting list was significantly shorter in patients in the NoKP group (*p* < 0.0001). The PELD score at LT was significantly higher in the NoKP group (*p* < 0.0001); however, when stratifying for age subgroups, significance was found only for patients transplanted between 2 and 3 years of age. The *Z*-score for weight at LT was not significantly different among the KP and NoKP groups (*p* = 0.39), and for all age subgroups. Similarly, the proportion of children with growth retardation at LT was not significantly different in both groups, as detected in 126 patients in the KP group (42.6%) and 40 patients in the NoKP group (41.2%) (*p* = 0.91). Total serum bilirubin levels at LT were significantly higher in the NoKP group (*p* < 0.0001); when stratifying for age subgroups, the corresponding difference in serum bilirubin levels was found only significant for patients transplanted between 2 and 3 years of age (*p* = 0.02). INR at LT was significantly higher in the NoKP group (*p* = 0.005); when stratifying for age subgroups, the corresponding difference was found only significant in patients transplanted between the age of 2 and 3 (*p* = 0.04), whereas there was a trend toward statistical significance for patients transplanted before the age of 1 (*p* = 0.07). Similarly, serum albumin levels at LT were significantly lower in the NoKP group (*p* = 0.0002), with a corresponding difference only found significant for patients in the 1–2-year age subgroup (*p* = 0.02). Portal vein hypoplasia was observed in 168 (56.8%) and 63 (64.9%) patients, in the KP and NoKP groups, respectively (*p* = 0.19), with no differences in age subgroups. Polysplenia syndrome was associated with BA in 41 patients in the KP group and 12 patients in the NoKP group (*p* = 0.86).

**Table 1:** Pre-LT clinical conditions estimation of 393 patients in the KP and NoKP groups.

		**KP group**	**NoKP group**	***p*-value**
**Number of patients**	**#**	**296**	**97**	
**Age at LT (years)**	**Median (IQR)**	**1.14 (0.76–2.08)**	**0.85 (0.65–1.30)**	** <0.0001**
**Time in waiting list (days)**	**Median (IQR)**	**78 (40–195)**	**48 (31–83)**	** <0.0001**
**PELD score**				
**All ages**	**Median (IQR)**	**12.9 (7.1–20.8)**	**18.0 (13.5–23.6)**	** <0.0001**
<1 y at LT		18.7 (12.3–26.5)	21.5 (16.6–23.4)	0.13
1–2 y at LT		11.8 (7.2–16.4)	11.8 (8.8–16.0)	0.47
2–3 y at LT		7.3 (2.7–12.9)	15.1 (10.1–26.8)	0.008
>3 y at LT		4.2 (0.3–9.0)	–	–
*ANOVA for trend*	*p-value*	* <0.0001*	*0.0011*	
***Z*****-score for weight at LT (SD)**				
**All ages**	**Median (IQR)**	**−1.7 (−2.7 to** **+1.8)**	**−1.7 (−6.7 to** **+1.8)**	**0.39**
<1 y at LT		−2.0 (−2.7 to −1.1)	−1.7 (−2.7 to −0.8)	0.22
1–2 y at LT		−1.8 (−2.9 to −0.6)	−1.4 (−3.4 to −0.8)	0.54
2–3 y at LT		−1.2 (−2.9 to −0.4)	−1.9 (−2.4 to −0.9)	0.65
>3 y at LT		−0.4 (−1.7 to +0.4)	–	–
*ANOVA for trend*	*p-value*	* <0.0001*	*0.51*	
**Total serum bilirubin (mg/dl)**				
**All ages**	**Median (IQR)**	**15.2 (7.8–20.2)**	**18.0 (13.4–23.8)**	** <0.0001**
<1 y at LT		16.8 (10.9–24.4)	17.7 (13.8–25.6)	0.16
1–2 y at LT		16.1 (10.7–19.2)	16.9 (11.5–20.8)	0.32
2–3 y at LT		13.8 (4.6–18.9)	20.8 (18.4–23.8)	0.02
>3 y at LT		6.1 (2.5–12.2)	–	–
*ANOVA for trend*	*p-value*	* <0.0001*	*0.31*	
**International normalized ratio**				
**All ages**	**Median (IQR)**	**1.20 (1.10–1.50)**	**1.34 (1.15–1.65)**	**0.005**
<1 y at LT		1.30 (1.10–1.87)	1.44 (1.22–1.84)	0.07
1–2 y at LT		1.20 (1.10–1.45)	1.20 (1.10–1.36)	0.97
2–3 y at LT		1.10 (1.00–1.21)	1.16 (1.10–2.58)	0.04
>3 y at LT		1.20 (1.10–1.38)	–	–
*ANOVA for trend*	*p-value*	*0.0002*	*0.49*	
**Serum albumin (gr/dl)**				
**All ages**	**Median (IQR)**	**3.20 (2.70–3.60)**	**2.80 (2.50–3.30)**	**0.0002**
<1 y at LT		2.90 (2.60–3.60)	2.90 (2.50–3.30)	0.14
1–2 y at LT		3.20 (2.70–3.50)	2.80 (2.30–3.25)	0.02
2–3 y at LT		3.40 (2.91–3.65)	2.85 (2.52–3.67)	0.39
>3 y at LT		3.50 (3.22–3.95)	–	–
*ANOVA for trend*	*p-value*	* <0.0001*	*0.71*	
**Portal vein hypoplasia**				
**All ages**	***# (%)***	***168 (56.8%)***	***63 (64.9%)***	**0.19**
<1 y at LT		*99 (75.0%)*	*45 (71.4%)*	0.60
1–2 y at LT		*50 (58.8%)*	*12 (48.0%)*	0.37
2–3 y at LT		*7 (23.3%)*	*5 (62.5%)*	0.08
>3 y at LT		*12 (24.5%)*	–	–
*Chi-square for trend*	*p-value*	* <0.0001*	*0.14*	

*Comparison between KP group vs. NoKP group is expressed in the right side of any row. The trend of variation among age subgroups within KP and NoKP groups is expressed at the bottom of the columns. KP, Kasai procedure (patient with previous Kasai operation); NoKP, No Kasai procedure (patients without previous Kasai operation); LT, liver transplantation; PELD, Pediatric end-stage liver disease; IQR, interquartile range; SD, standard deviation*.

**Figure 2:**
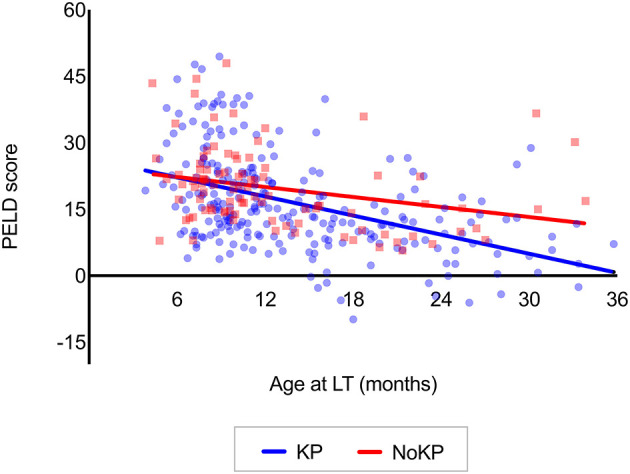
Correlation between the age at LT and PELD scores in patients transplanted before the age of 3 years (KP group: *R*^2^ = 0.21; *p* < 0.0001. NoKP group *R*^2^ = 0.08; *p* = 0.005. Difference between KP and NoKP slopes *p* = 0.04) (see Appendix A for further analyses). KP, Kasai procedure (patient with previous Kasai operation); NoKP, No Kasai procedure (patients without previous Kasai operation); LT, liver transplantation.

In the analysis of the trend of variation among age subgroups (Table 1, on the bottom of columns), the PELD score, total serum bilirubin levels, INR, albumin levels, and portal hypoplasia rate were all found to be progressively and significantly less severe in patients transplanted at older ages in KP. Conversely, only the PELD score showed a significant variation among age subgroups in the NoKP group, without a clearly progressive trend. In the correlation between age at LT and PELD score using linear regression, the PELD score was significantly lower at older ages, in both KP and NoKP groups (*p* < 0.0001 and *p* = 0.005, respectively); such variation over time was significantly more marked in KP groups when compared with NoKP (*p* = 0.04) (Figure 2). When using linear regression to correlate age at LT and the other pre-LT clinical variables, only for the KP group were less severe results found at older ages, except for serum albumin levels (Appendix A).

### Transplantation Data

Deceased-donor LT was performed in 121 patients (30.8%), of which 111 belonged to the KP group (91.7%). LDLT was performed in 272 patients (69.2%), of which 185 belonged to the KP group (68.0%). The use of LDLT was significantly more frequent in patients in the NoKP group (*p* < 0.0001). Portal hypoplasia has been managed using longitudinal portoplasty in 119 cases, jump graft in 64 cases, and direct anastomosis in 46 cases. A previously fashioned Roux-en-Y loop had to be revised in 94 cases (31.8% of KP patients).

### Overall Post-transplant Outcome

The overall 1 and 5-year patient survival rates were 96.9 and 94.4%, respectively. Causes of death were as follows: sepsis with multiorgan failure in nine patients (on days 2, 14, 29, 33, 71, 320, 425, 500, and 1,475 post-LT), primary non-function in two patients (on days 3 and 6 post-LT), miscellaneous complications after re-transplantation in five patients (on days 5, 254, 762, 1,540, and 2,277 post-LT), cerebral bleeding in one patient (on day 14), post-transplant lymphoproliferative disorders/lymphoma in three patients (on days 228, 521, and 1,299 post-LT), cardiac arrest in one patient (on day 576), chronic rejection in one patient (on day 3,662 post-LT), and unknown reasons in two patients (on days 5,305 and 7,975). Among these 24 deaths, 13 patients (54.2%) died within 1 year after LT (early mortality).

The overall 1- and 5-year graft survival rates were 93.4 and 90.9%, respectively. Twenty-two patients underwent re-transplantation for the following indications: hepatic artery thrombosis in five patients (on days 1, 4, 7, 9, and 10 post-LT), primary non-function in four patients (on days 2, 2, 3, and 6 post-LT), portal vein thrombosis in two patients (on days 3 and 30 post-LT), humoral rejection after ABO-incompatible LT in two cases (on days 93 and 130 post-LT), intrahepatic biliary strictures in two cases (on days 126 and 4,968 post-LT), and chronic rejection in seven patients (on days 210, 471, 537, 615, 1,484, 2,341, and 4,490 post-LT).

**Figure 3:**
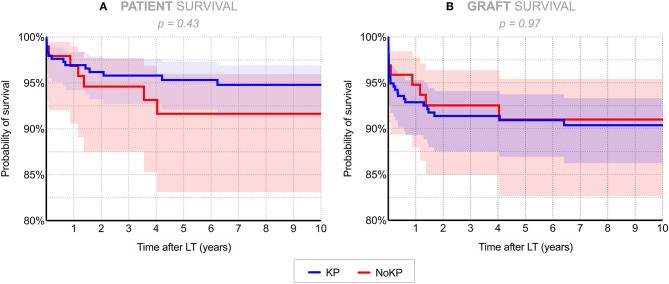
Overall **(A)** patient and **(B)** graft survival rates in 296 recipients after previous KP vs. 97 without KP (Kaplan–Meier analysis). Shaded areas represent 95% confidence intervals. KP, Kasai procedure (patient with previous Kasai operation); NoKP, No Kasai procedure (patients without previous Kasai operation).

One- and 5-year patient survival rates were 96.9 and 95.3% in the KP group, vs. 96.8 and 91.6% in the NoKP group, respectively (*p* = 0.43) (Figure 3A, Table 2), the corresponding figures for graft survival rates being 92.5 and 90.9% in the KP group, vs. 94.8 and 91.0% in the NoKP group (*p* = 0.97) (Figure 3B, Table 3).

**Table 2:** One- and 5-year post-LT patient survival rates in KP and NoKP, according to the age at LT.

	**KP group**		**NoKP group**		***p*-value**
	**1-year survival**	**5-year survival**	**1-year survival**	**5-year survival**	
**All ages**	**96.9%**	**95.3%**	**96.8%**	**91.5%**	**0.43**
<1 y	96.2%	94.5%	100%	98.0%	0.21
1–2 y	95.3%	92.1%	87.9%	76.7%	0.05
2–3 y	100%	100%	100%	100%	0.99
>3 y	100%	100%	–	–	–
*Log-rank for trend p-value*	*0.64*		*0.06*		

*Comparison between KP group vs. NoKP group is expressed in the right side of any row. The trend of variation among age subgroups within the KP and NoKP groups is expressed at the bottom of the columns. KP, Kasai procedure (patient with previous Kasai operation); NoKP, No Kasai procedure (patients without previous Kasai operation); LT, liver transplantation*.

**Table 3:** One- and 5-year graft survival rates in KP and NoKP, according to the age at LT.

	**KP group**		**NoKP group**		***p*-value**
	**1-year survival**	**5-year survival**	**1-year survival**	**5-year survival**	
**All ages**	**92.5%**	**90.9%**	**94.8%**	**91.0%**	**0.97**
<1 y	93.2%	91.5%	96.8%	96.8%	0.16
1–2 y	91.7%	89.9%	97.8%	76.5%	0.06
2–3 y	86.4%	86.4%	100%	100%	0.29
>3 y	97.9%	93.4%	–	–	–
*Log-rank for trend p-value*	*0.74*		*0.08*		

*Comparison between the KP group vs. NoKP groups is expressed in the right side of any row. The trend of variation among age subgroups within the KP and NoKP groups is expressed at the bottom of columns. KP, Kasai procedure (patient with previous Kasai operation); NoKP, No Kasai procedure (patients without previous Kasai operation); LT, liver transplantation*.

### Early Surgical Complications

The overall incidence of early (<45 days post-LT) surgical complications was significantly higher in the KP group (29.8%), when compared to the NoKP group (12.4%) (*p* = 0.01). Among these early surgical redos, abdominal complications were more frequent in the KP group, a difference which however only showed a tendency to statistical significance for abdominal complications in general (*p* = 0.06), and for bowel perforations in particular (*p* = 0.11). Early graft-related complications were significantly more frequent in the KP group (*p* = 0.005), namely, those related to the portal vein. When analyzing age subgroups, significance for higher portal vein complications in KP patients was established only for patients transplanted before the age of 1 year, while differences in other age subgroups did not reach statistical significance (data not shown). The number of surgical reoperations for each patient, as well as the impact of those complications on early mortality (within 1 year), overall mortality, and need for re-transplantation, was not significantly different among KP and NoKP groups (Table 4).

**Table 4:** Number of patients who developed at least one early surgical complication (occurred within 45 days after LT) in the KP and NoKP groups.

		**KP group**	**NoKP group**	***p*-value**
**Overall early complications**				
All ages	# (%)	88 (29.8%)	13 (13.4%)	0.01
<1 y at LT		36 (27.3%)	8 (12.7%)	0.03
1–2 y at LT		26 (30.6%)	4 (16.7%)	0.20
2–3 y at LT		6 (20.0%)	1 (12.5%)	0.99
*Chi-square for trend*	*p-value*	*0.69*	*0.83*	
**Early general complications**	# (%)	55 (18.5%)	10 (10.3%)	0.06
- Abdominal bleeding	# (%)	12 (4.1%)	4 (4.1%)	0.99
- Bowel perforation	# (%)	24 (8.1%)	3 (3.1%)	0.11
- Bowel occlusion	# (%)	3 (1.0%)	1 (1.0%)	0.99
- Other^*^	# (%)	14 (4.7%)	2 (2.1%)	0.38
**Early graft complications**	# (%)	47 (15.9%)	5 (5.6%)	0.005
- Hepatic artery dysfunction	# (%)	13 (4.4%)	2 (2.1%)	0.38
- Portal vein dysfunction	# (%)	14 (4.7%)	0 (0%)	0.03
- Outflow blockage	# (%)	2 (0.7%)	0 (0%)	0.99
- Biliary leakage	# (%)	16 (5.4%)	2 (2.1%)	0.26
- Biliary strictures	# (%)	6 (2.0%)	1 (1.0%)	0.99
Number of interventions/pts	Median (IQR)	1 (1–2)	1 (1–1)	0.24
Early mortality association	# (%^**^)	6 (6.8%)	1 (7.7%)	0.99
Overall mortality association	# (%^**^)	10 (11.4%)	1 (7.7%)	0.99
Re-transplantation association	# (%^**^)	13 (14.7%)	1 (7.7%)	0.69

*Comparison between KP group vs. NoKP group is expressed in the right side of any row*.

* *Including: non-proven bowel perforation, cleaning of intra-abdominal collections, etc*.

** *Those percentages are calculated with respect to the number of patients who developed an early surgical complication*.

*KP, Kasai procedure (patient with previous Kasai operation); NoKP, No Kasai procedure (patients without previous Kasai operation); LT, liver transplantation; IQR, interquartile range; pts, patients*.

### Overall Long-Term Surgical Graft Complications

The overall incidence of graft complications, including long-term vascular and biliary events, was non-significantly higher in the KP group (31.8%), when compared with the NoKP group (22.7%) (*p* = 0.09). When stratifying for any type of complications, no differences among groups have been found (Appendix B).

Hepatic artery dysfunction/thrombosis was detected in 15 cases; surgery has been attempted in 12 cases trying to restore adequate flow, which was effective in half of cases; re-transplantation has been needed in 5 cases; and three patients died.

Portal vein thrombosis has been a co-cause of early re-transplantation in two patients, including one patient with simultaneous outflow obstruction and consequent primary non-function, who died a few days after re-transplantation. Meso-Rex shunt has been performed in 15 patients, and meso-caval shunt has been used in one patient, due to non-patency of Rex' recessus; two of those patients died, at days 576 and 1,299 post-LT, respectively, due to unrelated causes. The rate of portal vein complication was non-significantly higher in patients with portal vein hypoplasia (*p* = 0.14); those patients with portal hypoplasia who underwent longitudinal portoplasty or venous jump graft had a non-significantly lower rate of portal vein complication, when compared with those who underwent direct anastomosis (*p* = 0.06 and *p* = 0.10, respectively).

Hepatic outflow dysfunction was detected in six patients: four were treated radiologically, one underwent surgery, and one underwent re-transplantation and died a few days later.

Among 61 patients who developed an early or late biliary anastomotic complication, 50 underwent surgical revision, including three patients who subsequently required interventional radiology treatment. Re-transplantation was needed in three patients, for related causes in two cases. Three patients died, including one after re-transplantation.

All those six patients who developed non-anastomotic biliary strictures underwent re-transplantation; one of them died afterward.

### Differences Among Transplant Eras

Among the total of 393 patients, 115 were transplanted during Era I (1993–1999), 115 during Era II (2000–2009), and 163 during Era III (2010–2018). The rate of patients transplanted without previous KP (NoKP group) significantly increased from 12.2% in Era I to 24.4% in Era II and 33.7% in Era III (*p* for trend < 0.0001). Similarly, the age at LT and the time on the waiting list increased over time (*p* for trend 0.04). The majority of pre-transplantation variables were found to be progressively more severe in the most recent transplant era, for both KP and NoKP groups; namely, the median PELD score was 13.2 during Era I, 12.2 during Era II, and 18.6 during Era III (*p* for trend 0.0002) (Appendix C).

The overall patient survival rate was not found to change significantly among patients transplanted during those three eras (*p* for trend 0.4348), but it was significantly lower in Era I (1-year survival 95.6%), when compared to both Era II (1-year survival 99.1%) and Era III (1-year survival 96.9%) (*p* = 0.02 and *p* = 0.02, respectively), without difference between the last two Eras (*p* = 0.43). No differences were found when analyzing KP and NoKP groups separately (Appendix D).

The overall and KP group graft survival rates changed significantly between eras (*p* for trend = 0.01 and 0.009, respectively), but no differences were found when comparing survivals during Era II and Era III (Appendix E).

The rate of overall early complications significantly reduced from 43.5% during Era I, to 21.7% during Era II and 15.9% during Era III (*p* for trend < 0.0001), and this was confirmed among each group separately, for both abdominal and graft complications. The rates of overall long-term vascular and biliary complications show a statistical tendency to decrease as well (*p* for trend = 0.09 and 0.05, respectively), particularly after Era I (Appendix F).

## Discussion

Back in 1985, Starzl and Gordon stated that “liver transplantation is made immeasurably more difficult by the performance of Kasai operation” (American College of Surgeons 1985 Annual Meeting) (11). According to this position of opinion leaders in the field, it would have been expected that LT might become the only primary treatment of BA. Actually, several factors contributed to maintaining the sequential strategy as the standard option for any infant who is eligible for the Kasai operation, including the following arguments (38): (1) the surgical exploration performed at the time of KP allows the definitive confirmation of BA by means of intra-operative cholangiography and liver biopsy; (2) some patients may survive a very long time with their native liver after an effective KP and may escape LT, sometimes until adolescence or even adulthood; (3) accordingly, successful KP could contribute to save organs in the context of worsening donor shortage; and (4) even in those children where KP does not provide reversal of cholestasis and in whom LT will be eventually required, partial biliary drainage may allow to buy months and sometimes years with prolonged preservation of general and nutritional status, contributing to reduce the risks of LT (39).

Data from single-center studies, as well as national or multicenter registries, reported that the 20-year native liver survival (NLS) rate after the Kasai operation ranges between 14 and 44% (12–17). The Japanese Biliary Atresia Registry published in 2003 and including 735 patients estimated a 5-year NLS rate between 52.7 and 64.5% (12). In Europe, the 1986–2009 French National Registry collecting 1,044 patients reported NLS rates at 40, 36, and 30%, at 5, 10, and 20 years, respectively (14). In England and Wales, BA patient care is centralized in only three supra-regional centers since 1999: among 424 patients registered until 2009, 5 and 10-year NLS rates were 46 and 40%, respectively (13). Accordingly, from these Japanese, French, and British data, it might be estimated that 389, 313, and 170 liver grafts, respectively, could be spared, a retrospective computation of interest in the context of organ shortage. Nevertheless, although such results suggest that Kasai operation still represents a good opportunity to avoid LT, long-term evolution with a native BA liver is often associated with assiduous lifelong cares, including management of recurrent cholangitis, portal hypertension, hepatopulmonary syndrome, portopulmonary hypertension, risk of malignant transformation, and eventually need for late LT (17, 26, 40). In a cohort of 63 patients who survived over 20 years with their native liver, all but two had clinical, sonographic, or histologic signs of cirrhosis, 44 had signs of portal hypertension, and 19 had late bacterial cholangitis (41).

Besides such BA patients who survive a long time with their native liver, the majority of children who underwent Kasai operation quickly develop signs of liver failure, eventually needing early transplantation before the age of 2 years. In a recent meta-analysis from Wang et al. including 1,560 BA patients who underwent LT, the mean age at LT after KP was 27 months (21). Similarly, in the most recent single-center series of LT for BA, LT was performed before the age of 1 year in 45–55% of children transplanted after the Kasai operation, the median age at LT ranging between 10 and 16 months (20, 22, 42). These data are consistent with our series, where 43.9% of LT after Kasai were carried out before the age of 1 year, the youngest patient being transplanted before the age of 4 months. Early transplantation in infants requires more challenging medical and surgical management: in a previous report from our group, including 328 BA recipients transplanted between 1984 and 2000, the best outcomes were achieved when transplantation was performed beyond the age of 6 years (43). On the other hand, the French registry reported that 15.6% of BA patients died without LT, confirming that these patients may develop rapidly progressing clinical deterioration and sometimes need semi-urgent LT (14).

In the present series, patients without previous Kasai operation had to be transplanted significantly earlier in life, a finding which could be expected considering the total absence of any biliary drainage in children without Kasai. Accordingly, using the PELD score as an indirect estimation of the pre-LT patient condition, the data of this work suggest that the clinical status at LT was significantly worse in children without previous Kasai, particularly in the 2–3-year age subgroup. Moreover, patients transplanted later in life after previous KP showed less severe pre-LT variables, when compared to those transplanted at younger ages. The putative benefit of KP to maintain some residual bile flow into the bowel, somehow contributing to improved absorption of lipids and liposoluble vitamins, might only be hypothesized. Other studies tried to investigate the relationship between Kasai operation, PELD score, and nutritional status before LT, reporting contradictory results. The PELD score at LT was found to be either non-significantly different (22) or significantly lower in patients after previous Kasai operation (19, 20, 44), but no study stratified patients according to their age category, as done in the present study. In this regard, it is essential to point out that the PELD score has been created to estimate the 3-month mortality risk for patients in the waiting list (24); consequently, its use for post-LT prognosis estimation should be interpreted with caution (45).

In the data presented in this work, post-LT graft and patient survival rates were not significantly different in children with or without previous Kasai operation. Some studies have already attempted to report the impact of KP on post-LT survival rates, the majority of them concluding that there were no significant differences (18, 21, 22). Conversely, other studies found that patients transplanted without previous Kasai had a lower mortality rate (23). It has been also shown that patients after Kasai who could be transplanted at older ages had better survival rates when compared to those who were transplanted either after early Kasai failure (<1 year of age) or without previous Kasai (19, 20). This last observation is not confirmed in our series, in which patients transplanted early after Kasai did not experience worse outcomes, and this is consistent with a further study which investigated only patients transplanted after Kasai, without finding any difference in survival rates according to the age at LT (42).

As previously identified by Starzl and Gordon, the risk for increased surgical morbidity after LT constitutes the main matter of concern following previous Kasai operation. This concern relates directly to the presence of sometimes dense intra-abdominal adhesions caused by prior abdominal surgery, their dissection at the time of native liver hepatectomy, and construction of biliary-digestive anastomosis being even further complicated by portal hypertension (21). In our study, we found that the rate of early reoperation was actually more frequent in patients with previous Kasai operation. Unexpectedly, the increased incidence of abdominal surgical complications in group KP showed only a statistical trend, namely, 18.5% after Kasai vs. 10.3% without Kasai (*p* = 0.06). Moreover, in the present series, the increased rate of early post-LT bowel perforation in the KP group did not reach statistical significance, namely, 8.1% after Kasai vs. 3.1% without Kasai (*p* = 0.11). These findings are to be compared to the sometimes conflicting observations from other studies, in which the rate of bowel perforation was reported to be significantly higher (20) or non-significantly different (18, 22) in patients with previous Kasai operation, ranging between 3.4 and 22%. The rate of redo surgeries for abdominal bleeding was 4.1% for both groups in our series, which is comparable with similar data from literature (18, 20). Regarding surgical complications related to the graft itself, a significantly increased rate of early portal vein complication was observed in the KP group, namely, 4.7% after Kasai vs. 0.0% without Kasai (*p* = 0.03). This result should be interpreted considering the equivalent proportion of children with portal vein hypoplasia at the time of LT (Table 1). The higher rate of portal vein complication might be explained by the inflammatory and fibrotic transformation of the liver pedicle in patients having undergone hilar dissection during KP. Whereas, such early surgical redos all added to the morbidity of LT, it should be noted that they did not impact on later graft and patient survival rates in either KP or NoKP groups (Table 4).

## Conclusions And Limitations

Results from the present study, including a large group of pediatric LT, support the strategy of sequential Kasai-then-transplantation in BA surgical management. Besides the sparing of organs, significantly better general status and older age at LT both constitute strong arguments to support such sequential strategy. Moreover, in the present series, the rate of bowel perforation post-LT was not found as statistically increased after previous Kasai operation.

Surgical management of BA is based on different types of organization worldwide. Traditionally, few pediatric surgery departments are involved in the LT program and, similarly, the majority of transplant surgeons do not perform Kasai operation. Accordingly, papers published from transplant teams tend to be focused on different aims, patients, and conclusions, when compared with those from pediatric surgeons. Such point represents a potential bias in interpreting the actual role of Kasai operation. The principal limitation of this work was indeed the absence of an intention-to-treat analysis starting at the time of decision to perform or not a Kasai operation. Accordingly, such analysis including children surviving in the long-term after KP with their native liver would contribute to better delineate the benefits to be expected from the sequential strategy, including for quality-of-life assessment.

## Data Availability Statement

The raw data supporting the conclusions of this article will be made available by the authors, without undue reservation.

## Ethics Statement

The studies involving human participants were reviewed and approved by Cliniques Universitaires Saint-Luc. Written informed consent from the participants' legal guardian/next of kin was not required to participate in this study in accordance with the national legislation and the institutional requirements.

## Author Contributions

RT conceived the study, contributed to data collection, and prepared the first draft of the manuscript. CdM contributed to data collection and interpretation. MS contributed to conceiving the study and to data collection. AC and VdM contributed to data collection. FS, AP, IS, XS, and ES contributed to interpretation of data. RR contributed to conceiving the study, to interpretation of data, and to revision of further drafts of the manuscript, before the final version. All authors contributed to critically revising the manuscript, approved the final version of the manuscript and the authorship list, and take full responsibility for the manuscript.

## Conflict of Interest

The authors declare that the research was conducted in the absence of any commercial or financial relationships that could be construed as a potential conflict of interest.
