# Supplementary material for: Sequential Treatment of Biliary Atresia With Kasai Hepatoportoenterostomy and Liver Transplantation: Benefits, Risks, and Outcome in 393 Children
Source: Front Pediatr. 2021 Jul 7;9:697581. doi: 10.3389/fped.2021.697581 (PMC8292612; doi:10.3389/fped.2021.697581)
Supplement: Supplementary file 1 [file Data_Sheet_1.pdf]

Supplementary Material

Sequential treatment of biliary atresia

**Appendix A.**<br>Correlation between age at LT and pre-LT clinical conditions in patients transplanted before the age of 3 years, using linear regression analysis.<br>R^2 measures the goodness of fit (associated p-value <0.05 indicates that slope is significantly non-zero).<br>In the last column, p value <0.05 estimates differences between KP and NoKP slopes.			
	**KP group**	**NoKP group**	***Difference between KP and NoKP slopes***
**Number of patients**	247	97	
**PELD score** (see Figure 2)	R^2 0.21<br>*(p <0.0001)*	R^2 0.08<br>*(p = 0.005)*	*p = 0.04*
**Z-score for weight at LT (SD)**	R^2 0.01<br>*(p = 0.07)*	R^2 0.01<br>*(p = 0.38)*	*p = 0.09*
**Total serum bilirubin (mg/dl)**	R^2 0.06<br>*(p <0.0001)*	R^2 0.01<br>*(p = 0.30)*	*p = 0.22*
**International Normalized Ratio**	R^2 0.06<br>*(p = 0.0002)*	R^2 0.01<br>*(p = 0.29)*	*p = 0.31*
**Serum albumin (gr/dl)**	R^2 0.005<br>*(p = 0.24)*	R^2 0.006<br>*(p = 0.44)*	*p = 0.20*

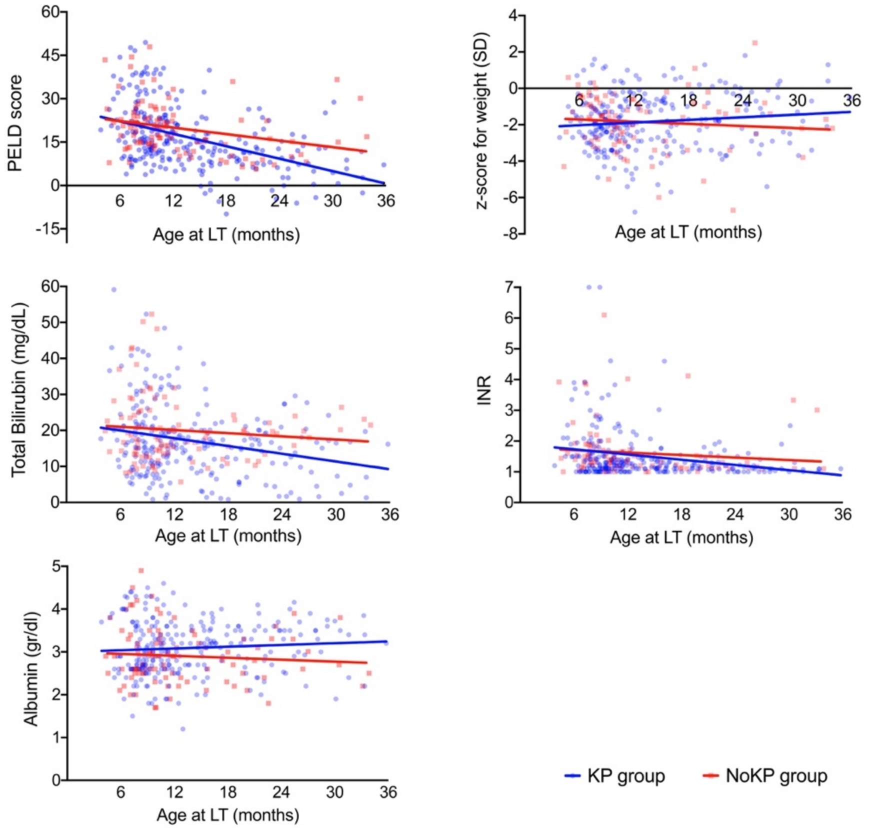

Abbreviations: LT: liver transplantation; KP: Kasai procedure (patient with previous Kasai operation); NoKP: No Kasai procedure (patients without previous Kasai operation); PELD: Pediatric end-stage liver disease; INR: International Normalized Ratio; SD: standard deviation.

**Appendix B.** Overall surgical graft complications: comparison between KP and NoKP groups.				
		**KP group**	**NoKP group**	*p-value*
**Total graft complications**				
all ages	# (%)	94 (31.8%)	22 (22.7%)	*0.10*
< 1y at LT		41 (31.1%)	15 (23.8%)	*0.32*
1-2y at LT		30 (35.3%)	5 (20.0%)	*0.22*
2-3y at LT		6 (20.0%)	1 (12.5%)	*0.99*
Chi-square for trend	*p-value*	*0.53*	*0.45*	
Hepatic artery thrombosis	# (%)	12 (4.1%)	3 (3.1%)	*0.99*
Portal vein thrombosis	# (%)	30 (10.1%)	7 (7.2%)	*0.55*
Outflow blockage	# (%)	6 (2.0%)	0 (0%)	*0.34*
Anastomotic biliary stricture	# (%)	50 (16.9%)	12 (12.4%)	*0.34*
Non-anastomotic biliary strictures	# (%)	6 (2.0%)	0 (0%)	*0.34*

Abbreviations: LT: liver transplantation; KP: Kasai procedure (patient with previous Kasai operation); NoKP: No Kasai procedure (patients without previous Kasai operation).

Appendix C.
Pre-LT clinical conditions estimation according to transplant eras.

		**ERA I** (1993-1999)	**ERA II** (2000-2009)	**ERA III** (2010-2018)	*p-value for trend*
**Number of patients**	# (%)				
**Overall**		**115**	**115**	**163**	
KP group		101 (87.8%)	87 (75.6%)	108 (66.3%)	
NoKP group		14 (12.2%)	28 (24.4%)	55 (33.7%)	*<0.0001*
**Age at LT (years)**	Median (IQR)				
**Overall**		**1.17 (0.75-2.27)**	**0.92 (0.73-1.78)**	**1.00 (0.71-1.75)**	***0.04***
KP group		1.27 (0.75-2.43)	1.05 (0.74-2.08)	1.08 (0-79-1.83)	*0.17*
NoKP group		0.79 (0.61-1.40)	0.85 (0.60-1.09)	0.90 (0.65-1.50)	*0.59*
**Time on waiting list (days)**	Median (IQR)				
**Overall**		**105 (36-236)**	**73 (37-230)**	**58 (37-87)**	***0.006***
KP group		112 (44-248)	110 (40-302)	60 (37-91)	*0.04*
NoKP group		39 (19-146)	38 (31-69)	58 (37-86)	*0.76*
**PELD score**	Median (IQR)				
**Overall**		**13.15 (8.65-18.01)**	**12.24 (7.15-19.22)**	**18.58 (9.39-26.51)**	***0.0002***
KP group		12.81 (8.11-17.90)	10.45 (5.94-18.27)	16.20 (7.25-25.48)	*0.02*
NoKP group		15.59 (11.80-20.65)	16.10 (11.97-22.35)	20.71 (14.98-26.92)	*0.03*
**Z-score for weight at LT (SD)**	Median (IQR)				
**Overall**		**-2.1 (-3.2 - -0.9)**	**-1.3 (-2.4 - -0.3)**	**-1.7 (-2.5 - -0.7)**	***0.03***
KP group		-2.1 (-3.0 - -0.8)	-1.1 (-2.3 - -0.2)	-1.7 (-2.6 - -0.7)	*0.004*
NoKP group		-2.3 (-4.5 - -1.2)	-1.75 (-2.85 - -0.8)	-1.5 (-2.4 - -0.8)	*0.013*
**Total serum bilirubin (mg/dl)**	Median (IQR)				
**Overall**		**16.0 (10.0-20.6)**	**14.3 (11.4-22.9)**	**17.3 (11.4-22.9)**	***0.06***
KP group		16.0 (9.8-20.5)	12.5 (6.7-17.2)	16.8 (7.0-22.0)	*0.80*
NoKP group		16.4 (10.9-26.4)	16.8 (13.9-23.3)	19.1 (14.2-24.0)	*0.96*
**International Normalized Ratio**	Median (IQR)				
**Overall**		**1.2 (1.0-1.4)**	**1.2 (1.1-1.4)**	**1.4 (1.2-1.9)**	***<0.0001***
KP group		1.2 (1.0-1.4)	1.2 (1.1-1.4)	1.3 (1.1-1.9)	*0.0008*
NoKP group		1.2 (1.0-1.5)	1.2 (1.1-1.4)	1.5 (1.2-1.9)	*0.02*
**Serum albumin (gr/dl)**	Median (IQR)				
**Overall**		**3.5 (3.0-3.9)**	**3.2 (2.8-3.5)**	**2.7 (2.5-3.2)**	***<0.0001***
KP group		3.5 (3.0-3.9)	3.2 (2.8-3.6)	2.8 (2.5-3.4)	*<0.0001*
NoKP group		3.5 (3.1-4.0)	3.0 (2.5-3.4)	2.6 (2.4-2.9)	*<0.0001*
**Portal vein hypoplasia**	# (%)				
**Overall**		**67 (58.3%)**	**70 (60.9%)**	**94 (57.7%)**	***0.88***
KP group		56 (55.4%)	52 (59.8%)	60 (55.6%)	*0.99*
NoKP group		11 (78.6)	18 (64.3%)	34 (81.8%)	*0.25*

Abbreviations: LT: liver transplantation; KP: Kasai procedure (patient with previous Kasai operation); NoKP: No Kasai procedure (patients without previous Kasai operation); PELD: Pediatric end-stage liver disease; INR: International Normalized Ratio; SD: standard deviation.

Appendix D.
Overall patient survival rate according to transplant eras.

	ERA I (1993-1999)		ERA II (2000-2009)		ERA III (2010-2018)		*p for trend*	*p-value*		
	1-year	5-year	1-year	5-year	1-year	5-year		*I vs II*	*I vs III*	*II vs III*
**Overall**	**95.6%**	**92.0%**	**99.1%**	**97.3%**	**96.9%**	**94.0%**	***0.43***	***0.07***	***0.42***	***0.20***
KP group	95.0%	93.0%	98.8%	97.7%	97.2%	94.8%	*0.29*	0.15	0.34	0.43
NoKP group	100%	84.6%	100%	96.3%	100%	92.4%	0.85	0.18	0.86	0.28

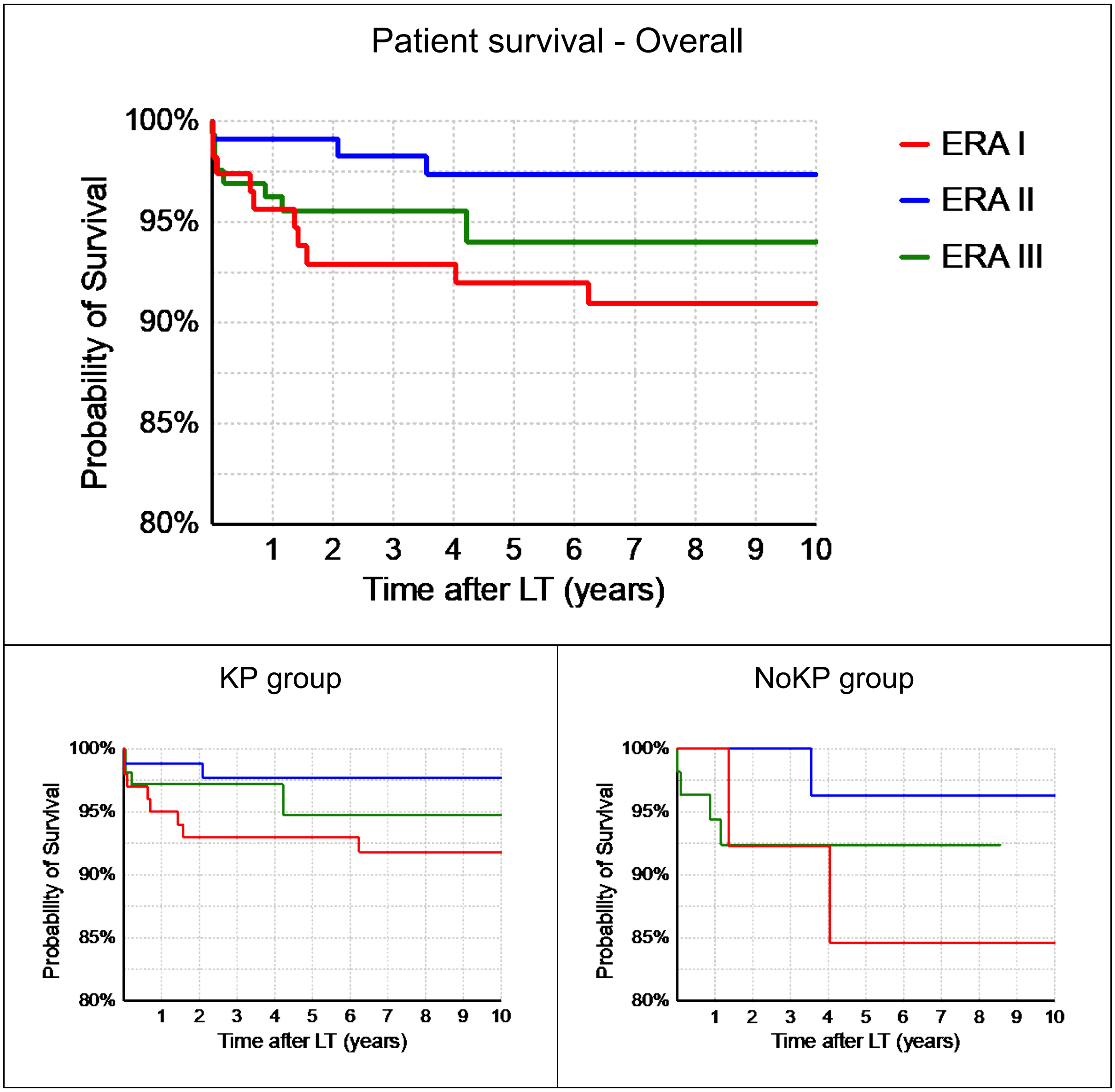

Abbreviations: LT: liver transplantation; KP: Kasai procedure (patient with previous Kasai operation); NoKP: No Kasai procedure (patients without previous Kasai operation).

Appendix E.
Overall graft survival rate according to transplant eras.

	ERA I (1993-1999)		ERA II (2000-2009)		ERA III (2010-2018)		*p for trend*	*p-value*		
	1-year	5-year	1-year	5-year	1-year	5-year		*I vs II*	*I vs III*	*II vs III*
**Overall**	**87.8%**	**84.1%**	**96.5%**	**94.8%**	**95.0%**	**92.9%**	***0.01***	***0.02***	***0.02***	***0.73***
KP group	86.1%	84.0%	96.5%	95.4%	95.3%	93.1%	*0.009*	*0.01*	*0.02*	*0.76*
NoKP group	100%	84.6%	92.9%	92.9%	94.4%	92.4%	*0.75*	*0.77*	*0.57*	*0.97*

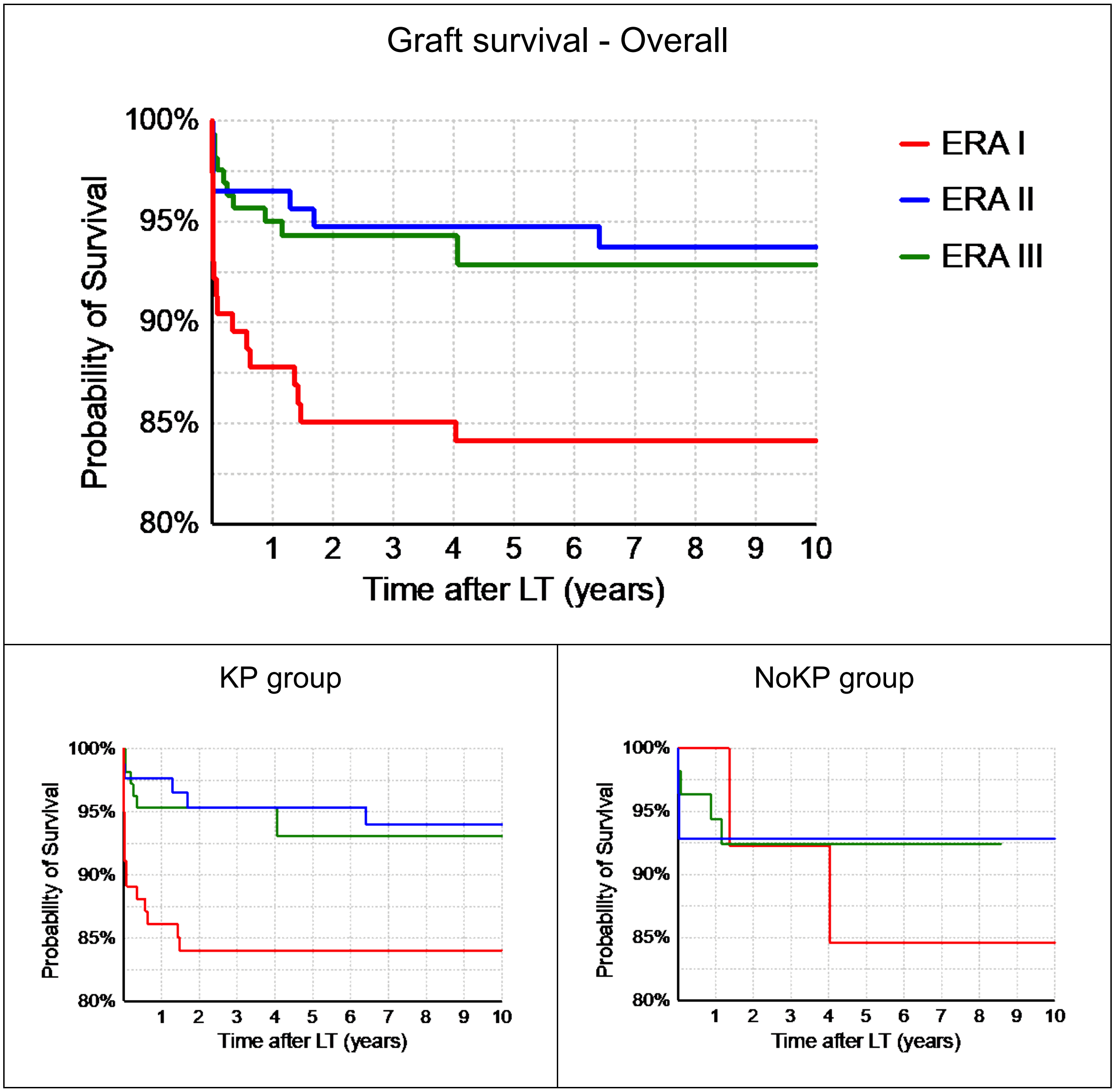

Abbreviations: LT: liver transplantation; KP: Kasai procedure (patient with previous Kasai operation); NoKP: No Kasai procedure (patients without previous Kasai operation).

Appendix F.

Early (occurred within 45 days after LT) and overall surgical complications according to transplant eras.

		**ERA I** (1993-1999)	**ERA II** (2000-2009)	**ERA III** (2010-2018)	*p-value for trend*
**Number of patients**					
**Overall**	# (%)	**115**	**115**	**163**	
KP group		101 (87.8%)	87 (75.6%)	108 (66.3%)	
NoKP group		14 (12.2%)	28 (24.4%)	55 (33.7%)	*<0.0001*
**Overall early complications**					
**Overall**	# (%)	**50 (43.5%)**	**25 (21.7%)**	**26 (15.9%)**	*<0.0001*
KP group		45 (44.5%)	21 (24.1%)	22 (20.4%)	*0.0001*
NoKP group		5 (35.7%)	4 (14.3%)	4 (7.3%)	*0.008*
**Early abdominal complications**					
**Overall**	# (%)	**33 (28.7%)**	**19 (16.5%)**	**13 (8.0%)**	***<0.0001***
KP group		28 (27.7%)	17 (19.5%)	10 (9.3%)	*0.0006*
NoKP group		5 (35.7%)	2 (7.1%)	3 (5.5%)	*0.004*
**Early graft complications**					
**Overall**	# (%)	**25 (21.7%)**	**9 (7.8%)**	**18 (11.0%)**	***0.02***
KP group		24 (23.8%)	7 (8.0%)	16 (14.8%)	*0.08*
NoKP group		1 (7.1%)	2 (7.1%)	2 (3.6%)	*0.48*
**Overall graft vascular compl.**					
**Overall**	# (%)	**20 (17.4%)**	**18 (15.7%)**	**17 (10.4%)**	***0.09***
KP group		19 (18.8%)	11 (12.6%)	15 (13.9%)	*0.33*
NoKP group		1 (7.1%)	7 (25.0%)	2 (3.6%)	*0.15*
**Overall graft biliary compl.**					
**Overall**	# (%)	**27 (23.5%)**	**16 (13.9%)**	**23 (14.1%)**	***0.05***
KP group		24 (23.8%)	12 (13.8%)	18 (16.7%)	*0.19*
NoKP group		3 (21.4%)	4 (14.3%)	5 (9.1%)	*0.19*

Abbreviations: LT: liver transplantation; KP: Kasai procedure (patient with previous Kasai operation); NoKP: No Kasai procedure (patients without previous Kasai operation).
